# Postoperative Buttock Skin Injuries Not Explained by Electrosurgical Burns: Three Cases Suggesting an Ischemia–Reperfusion Mechanism

**DOI:** 10.3390/jcm15062093

**Published:** 2026-03-10

**Authors:** Hiroshi Tanabe, Yoshinori Nakamura

**Affiliations:** 1 Department of Dermatology, Tenri Hospital, Tenri 632-8552, Nara, Japan; 2 Department of Medical Home Healthcare Center, Tenri Hospital Shirakawa Branch, Tenri 632-0003, Nara, Japan; yoshi_nakamura@mue.biglobe.ne.jp

**Keywords:** postoperative buttock skin injuries, electrosurgical burns, stray high-frequency current, deep tissue injury, ischemia–reperfusion injury

## Abstract

Postoperative buttock skin lesions are uncommon complications that can cause severe pain and delayed healing. While often attributed to pressure, some clinical reports have classified them as electrosurgical burns. However, the electrophysical plausibility of this attribution under standard operating conditions is uncertain. We present three cases of buttock skin lesions appearing on the first postoperative day with severe pain and evidence of underlying muscle involvement. In each case, operative conditions, device usage, and clinical findings were inconsistent with typical electrosurgical injury patterns. These cases suggest that some postoperative buttock skin lesions may represent ischemia–reperfusion-related deep tissue injury rather than thermal injury. Given the limited sample size, this report is hypothesis-generating. Prospective studies are needed to clarify the roles of perioperative ischemic and mechanical factors in these lesions.

## 1. Introduction

Postoperative buttock skin injury is an uncommon but clinically significant complication characterized by painful erythematous lesions that typically appear on the first postoperative day. These lesions may involve underlying deep tissue damage and require prolonged healing [1]. In dermatology, this condition has been termed “post-spinal anesthesia erythema,” though its etiology remains unclear [1]. Clinically, these lesions may be misdiagnosed as contact dermatitis, low-temperature burns, or superficial pressure injuries.

In Japan, several dermatological reports have attributed postoperative buttock skin lesions to electrosurgical burns, based primarily on case series by Hayashi et al. [2] and Hattori et al. [3]. However, this mechanism lacks consistent electrophysical support. Electrical engineering analyses suggest that while electrosurgical burns may occur under exceptional circumstances, standard use of devices conforming to Japanese Industrial Standards (JIS) is unlikely to generate sufficient dispersed current to cause extensive skin injury [4]. The etiology of these lesions therefore remains debated [5]. In rare cases, severe gluteal muscle ischemia may progress to gluteal compartment syndrome [6], and medico-legal case reports have further highlighted the clinical and institutional implications of potential misattribution of postoperative buttock lesions to electrosurgical burns [7].

Since deep tissue injury (rathe) was incorporated into pressure injury classification systems, alternative mechanisms have been proposed. DTI is defined as pressure-related damage to underlying soft tissues beneath intact or discolored skin, often presenting with localized pain or discoloration [8,9]. Recent pathophysiological studies highlight the role of ischemia–reperfusion processes in DTI development. Surgical mechanical loading may induce localized deep tissue ischemia, and subsequent reperfusion can trigger oxidative stress, inflammation, and delayed tissue damage beneath intact skin [10,11,12]. These lesions often appear hours postoperatively and may be accompanied by elevated muscle-derived enzymes including creatine kinase (CK), aspartate aminotransferase (AST), and lactate dehydrogenase (LDH), indicating muscle involvement [11]. Ultrasound-based assessment has also been used to evaluate deep tissue changes in pressure-related injury contexts [13].

This report presents three cases of postoperative buttock skin injury occurring under conditions inconsistent with electrosurgical burns and discusses ischemia–reperfusion-related deep tissue injury as a plausible alternative mechanism.

## 2. Case Reports

We present three cases of postoperative buttock skin injuries that challenge the electrosurgical burn hypothesis. These cases were retrospectively identified from patients referred to dermatology through the institutional pressure ulcer committee after postoperative buttock skin lesions were reported. The three cases occurred at different times (April 2004, December 2005, and May 2010) and were selected for their shared clinical features and diagnostic relevance.

Case 1: Postoperative Buttock Skin Injury Associated with a Nonconductive Warming Blanket

An 80-year-old woman underwent elective coronary artery bypass grafting under general anesthesia with an operative time of approximately 8 h. At discharge from the operating room, her buttock skin appeared normal.

**Figure 1:**
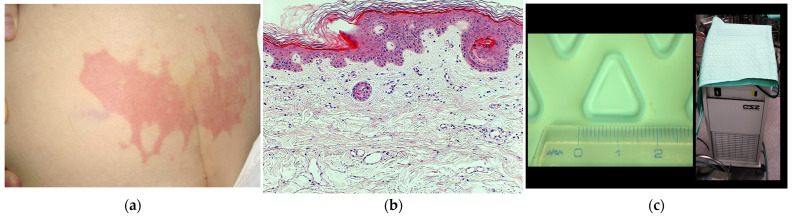
Case 1: Postoperative buttock skin lesion. (**a**) Clinical photograph at initial consultation on postoperative day 1 showing a well-demarcated erythematous lesion (22 × 5 cm) in the sacral region with severe localized pain. The erythema showed a mesh-like pattern of equilateral triangles with approximately 1.5 cm sides. (**b**) Histopathology of punch biopsy from the erythematous area on postoperative day 2. H&E staining (×100) showed intact epidermis and dermis without necrosis, blister formation, or inflammatory infiltration. Mild dermal capillary dilation was observed. (**c**) Circulating warm-water blanket device used during surgery (Blanketrol II; Cincinnati Sub-Zero, Cincinnati, OH, USA) (**right**) and a magnified view of the blanket surface (**left**). Warm water (41 °C) circulated through embossed equilateral triangular projections (approximately 1.5 cm per side). A nonconductive moisture-absorbing waterproof sheet was placed between the patient and the device.

On postoperative day 1, she developed severe right buttock pain. Physical examination revealed a well-demarcated erythematous lesion (22 × 5 cm) in the sacral region with a mesh-like pattern (Figure 1a). Serum CK was markedly elevated, peaking at 2448 U/L.

Punch biopsy on postoperative day 2 showed intact epidermis and dermis without necrosis or inflammation (Figure 1b). The lesion configuration corresponded to the area covered by the intraoperative circulating-water warming blanket (Figure 1c).

Conservative management with pressure relief and analgesia was initiated. The erythema gradually resolved over two weeks, leaving mild post-inflammatory hyperpigmentation without ulceration or scarring.

Case 2: Postoperative Buttock Skin Injury Following Bipolar Electrocautery Use

A 15-year-old girl underwent anterior cruciate ligament reconstruction under combined spinal-epidural anesthesia. Operative duration was approximately 1 h 45 min, with a total operating room time of 2 h 30 min. She remained supine throughout. At discharge from the operating room, buttock skin appeared normal.

**Figure 2:**
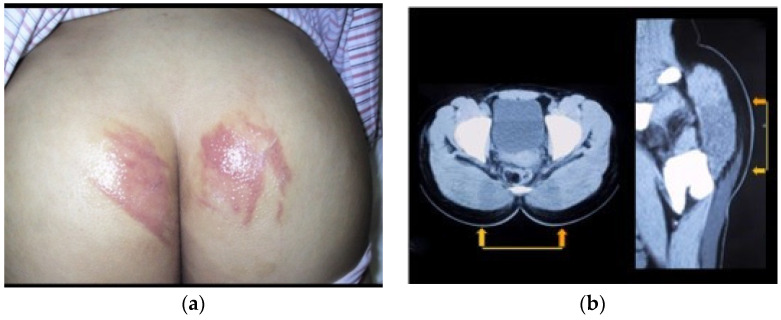
Case 2: Postoperative buttock skin lesion. (**a**) Clinical photograph on postoperative day 1 showing diffuse bilateral buttock swelling. A linear erythematous lesion was observed on the right buttock, and a band-like erythematous lesion on the left. Lesions were non-blanchable on glass-plate compression. Severe pain prevented supine positioning. (**b**) Axial computed tomography image on postoperative day 2 showing localized edema in the buttock subcutaneous tissues and bilateral gluteal muscles adjacent to the sacrum (arrows).

On postoperative day 1, she developed severe bilateral buttock pain with irregularly shaped erythema and diffuse gluteal swelling (Figure 2a). Pain was severe enough to prevent supine positioning. Laboratory testing revealed markedly elevated muscle enzymes: CK, 6075 U/L; AST, 321 IU/L; and LDH, 511 IU/L. Computed tomography showed diffuse gluteal muscle edema, predominantly in the dorsal pelvic and sacral regions, without soft-tissue infection or fluid collection (Figure 2b).

An epidural catheter was reinserted for pain control. The skin lesions gradually improved over two weeks. Transient purpuric discoloration developed during recovery and resolved, leaving mild residual scarring.

Case 3: Postoperative Buttock Skin Injury Without Electrocautery Use

An 87-year-old woman underwent internal fixation for right trochanteric fracture under general anesthesia. Operative duration was approximately 55 min, with total operating room time of 2 h 15 min. She was positioned supine with lower limb traction. No monopolar or bipolar electrocautery was used. At discharge from the operating room, buttock skin appeared normal.

**Figure 3:**
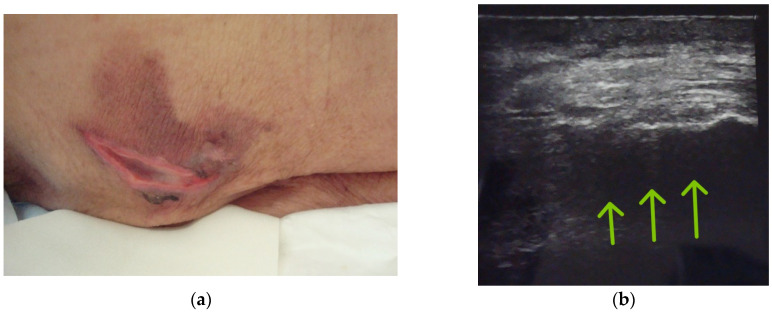
Case 3: Postoperative buttock skin lesion. (**a**) Clinical photograph of the sacral region on postoperative day 1 showing a painful erythematous lesion (6.0 × 7.7 cm) with central ulcerative necrosis. No abnormalities were noted at operating room discharge. (**b**) Postoperative ultrasonography showing heterogeneous echogenicity predominantly in the gluteal muscle layer (arrows), consistent with muscle edema.

On postoperative day 1, she developed a painful erythematous lesion (6.0 × 7.7 cm) in the left sacral region, which had been non-weight-bearing during surgery. A central crescent-shaped ulcerative necrosis (approximately 5.5 × 1.0 cm) was observed. CPK was mildly elevated, increasing from 25 U/L preoperatively to 61 U/L on postoperative day 1 (Figure 3a).

Postoperative ultrasonography revealed heterogeneous echogenicity within the gluteal muscle layer, consistent with muscle edema (Figure 3b) [13]. Conservative management was initiated. The surrounding erythema resolved within approximately two weeks, while complete healing of the central ulcer required over 3 months.

## 3. Discussion

### 3.1. Electrosurgical Burn Hypothesis Reconsidered

Postoperative buttock skin lesions have occasionally been attributed to stray radiofrequency currents during electrosurgical procedures. However, under standard operating conditions, monopolar electrosurgical units are designed to limit leakage currents according to safety standards such as JIS T 0601-1 [14]. Thermal injury typically requires highly localized current concentration rather than broad dispersion. When electrical current is distributed across a wide tissue surface, the resulting current density is generally insufficient to generate clinically significant heat, as predicted by the Joule heating equation (*Q* = *I*^2^·*R*·*t*) [15]. Additional electrophysical considerations are summarized in Supplementary File S1.

### 3.2. Clinical Counterexamples to the Electrosurgical Burn Hypothesis

Previous dermatological reports have suggested that postoperative gluteal lesions represent electrosurgical burns based primarily on temporal and anatomical observations [2,3]. However, these interpretations lack direct electrophysical validation. In the present cases, several operative circumstances were incompatible with an electrosurgical burn mechanism: (i) lesion morphology corresponding to a nonconductive warming device (Case 1); (ii) occurrence following exclusive use of bipolar electrocautery (Case 2); and (iii) lesion development without any electrosurgical device use (Case 3). In all cases, buttock skin was intact at operating room discharge, and painful lesions developed with delayed onset on postoperative day 1. These findings suggest alternative mechanisms should be considered when lesion morphology, operative conditions, and temporal patterns are inconsistent with focal current concentration.

### 3.3. Differential Diagnosis and Temporal Characteristics

Several mechanisms should be considered in the differential diagnosis of postoperative buttock skin lesions. Positioning-related muscle injury from prolonged pressure may cause localized deep tissue ischemia, particularly in anesthetized patients [9]. Perioperative microvascular vulnerability associated with hypotension, vasoconstrictive agents, or impaired perfusion may further increase susceptibility to ischemic injury [10]. Non-electrical thermal injuries from warming devices or chemical preparation agents should also be considered, though these typically produce earlier, more superficial manifestations.

Unlike thermal burns, which usually manifest immediately after exposure, all three cases demonstrated delayed erythema onset approximately 12–24 h postoperatively. Such delayed presentation is characteristic of ischemia–reperfusion-related tissue injury [11,12]. Previous perioperative studies have similarly reported that sacral or gluteal skin injuries first recognized one to two days post-surgery were inconsistent with electrosurgical burns and instead represented pressure-related or deep tissue injury [16].

### 3.4. Ischemia-Reperfusion-Related Deep Tissue Injury as a Plausible Mechanism

The clinical findings observed in these cases, including severe deep pain disproportionate to cutaneous findings, elevated muscle-associated enzymes, and imaging evidence of gluteal muscle involvement, are compatible with ischemia–reperfusion-related deep tissue injury [11,17]. Experimental and clinical studies have demonstrated that pressure-induced ischemia followed by reperfusion generates reactive oxygen species, endothelial injury, and inflammatory cascades, leading to muscle damage beneath intact skin [11,12,18,19]. While these observations do not establish definitive etiology, they suggest ischemia–reperfusion injury may be a clinically relevant mechanism in selected postoperative buttock lesions.

Because the present observations are derived from a small case series, the proposed mechanism should be interpreted as a hypothesis-generating observation rather than a definitive causal conclusion. Further prospective studies are needed to clarify the relative contribution of ischemia–reperfusion processes in postoperative buttock skin lesions. A broader pathophysiological framework supporting this interpretation is summarized in Supplementary File S2.

### 3.5. Clinical and Institutional Implications

Accurate differentiation between electrosurgical burns and ischemia-related deep tissue injury has important clinical and institutional implications. Misattribution of postoperative lesions to electrosurgical injury may lead to inappropriate management, unnecessary medico-legal concerns, or misdirected incident investigations [7,20]. Careful evaluation of operative conditions, lesion distribution, and temporal onset combined with interdisciplinary collaboration among surgical, anesthesiology, dermatology, and clinical engineering teams may improve diagnostic accuracy and perioperative risk management. Further prospective studies are needed to clarify the relative contribution of these mechanisms. Comparable ischemia–reperfusion mechanisms have been described in related entities such as gluteal compartment syndrome and coma blisters [21,22].

This report is not intended to replace existing diagnostic interpretations but to provide an additional pathophysiological perspective that may assist multidisciplinary evaluation of postoperative buttock skin lesions.

## 4. Conclusions

This report describes three postoperative buttock skin lesions unlikely to be explained by electrosurgical burns under standard operating conditions. The clinical course, lesion characteristics, and available laboratory and imaging findings were collectively more consistent with ischemia–reperfusion-related deep tissue injury. These hypothesis-generating observations suggest that selected postoperative buttock lesions traditionally attributed to electrosurgical burns may, in certain clinical contexts, represent ischemia–reperfusion–associated injury. Further systematic investigation is required to better define the epidemiology, risk factors, and preventive strategies for this condition. Careful multidisciplinary evaluation may help improve diagnostic accuracy when postoperative buttock lesions are encountered.

## Data Availability

The original contributions presented in this study are included in the article/Supplementary Material. Further inquiries can be directed to the corresponding author.

## Supplementary Materials

The following supporting information can be downloaded at: https://www.mdpi.com/article/10.3390/jcm15062093/s1, Supplementary File S1: Electrophysical Considerations; Supplementary File S2: Ischemia–Reperfusion Framework. References [23,24,25] are cited in the Supplementary Materials.
