# Supplementary material for: Postoperative Buttock Skin Injuries Not Explained by Electrosurgical Burns: Three Cases Suggesting an Ischemia–Reperfusion Mechanism
Source: J Clin Med. 2026 Mar 10;15(6):2093. doi: 10.3390/jcm15062093 (PMC13026345; doi:10.3390/jcm15062093)
Supplement: Supplementary file 1 [file jcm-15-02093-s001.zip › jcm-4141982-supplementary.pdf]

Supplementary File S1

Electrical Engineering Considerations Regarding the Electrosurgical Burn Hypothesis

Biophysical Analysis of Electrosurgical Burns in Relation to Postoperative Buttock Lesions

S1, 1. Basics of Electrosurgical Units

Electrosurgical units (ESUs) used in modern surgery typically operate at frequencies of 300 kHz to 5 MHz with power outputs of 30–300 W. In monopolar configurations, electrical current flows from the active electrode (e.g., scalpel tip) through the patient's body and returns via a dispersive return electrode placed on a broad skin surface such as the thigh or back. This design concentrates energy at the surgical site while dispersing return current over a large area [4].

In contrast, bipolar electrosurgery confines current to tissue between two closely spaced electrodes. Bipolar systems do not require a dispersive return electrode and do not permit current to traverse distant body regions. Consequently, current dispersion to remote tissues such as the buttocks is unlikely under standard operating conditions [23].

S1, 2. Joule Heating and Its Limitations

Thermal injury from electrosurgery results from Joule heating, governed by [4] [23]:

Joule heating equation:

$$Q = I^2 \; R \; t$$

Where:

- Q = heat generated (joules)
- I = electric current (amperes)
- R = electrical resistance (ohms)
- t = duration of current application (seconds)

Example calculation (assuming $I = 0.15$ A, $R = 20\ \Omega$, and $t = 600$ s): $Q = (0.15)^2 \times 20 \times 600 = 270$ J

Even under conservative assumptions, the heat generated is generally insufficient to produce clinically significant thermal injury when current is dispersed over a large anatomical area.

S1, 3. Current Density and Localization of Electrosurgical Burns

Current density (J) is defined as:

$J = I / A$

Where:

J = current density

I = electric current

A = contact area of the electrode

This principle explains why electrosurgical burns typically occur at the active electrode tip, where contact area is extremely small and current density is high [4] [23]. At dispersive interfaces, such as return electrodes or large body surfaces like the buttocks, the contact area is extensive, resulting in markedly lower current density and reduced heat generation.

S1, 4. Skin Effect at High Frequencies

At frequencies exceeding approximately 1 MHz, the skin effect becomes prominent, whereby alternating current preferentially flows near the surface of a conductor. This phenomenon enhances localized surface heating at narrow metallic structures such as surgical blades.

However, the skin effect primarily causes superficial heating at narrow conductive interfaces and is unlikely to account for deep muscular injury distributed over a broad anatomical region. Injuries localized to deep gluteal muscle without corresponding epidermal necrosis are therefore unlikely to be explained by skin-effect–mediated thermal injury [4] [23].

S1, 5. Clinical Considerations and Limitations of the Electrosurgical Burn Hypothesis

The assumption that postoperative gluteal erythema represents electrosurgical burns may contribute to diagnostic uncertainty and medico-legal concern. Several considerations are relevant when evaluating this hypothesis:

- ESUs are subject to strict safety regulations, including current limits defined by standards such as JIS T06014 [24].
- Burns from dispersive electrodes typically require device malfunction, electrode detachment, or highly localized conductive contact.
- Delayed erythema onset, often appearing 12–24 h postoperatively, is generally inconsistent with thermal burns, which typically manifest shortly after exposure [4] [23].

The observed clinical course in the present cases is compatible with deep tissue injury from intraoperative immobility, pressure-related ischemia, and subsequent reperfusion injury. This interpretation is consistent with both the temporal pattern and morphological characteristics of the lesions.

S1, 6. Conclusion

- Stray current from properly functioning ESUs is unlikely to generate sufficient heat to injure gluteal tissues under standard operating conditions.
- Electrosurgical burns typically occur at sites of high current density, such as active electrode tips or focal conductive contact points.
- Delayed-onset postoperative gluteal lesions may, in selected clinical contexts, be more plausibly explained by ischemia–reperfusion–related deep tissue injury from intraoperative pressure and immobilization.

The hypothesis that monopolar electrosurgical burns cause postoperative gluteal erythema is not well supported under standard operating conditions. Accurate identification of the underlying mechanism is important for appropriate diagnosis, clinical education, and institutional risk assessment.

Supplementary File S2

Deep Tissue Injury (DTI) as a Potential Ischemia–Reperfusion Phenomenon: A Pathophysiological Framework

S2, 1. Introduction: Beyond Pressure—Toward an Expanded Pathophysiological Framework

This supplementary file provides a pathophysiological framework to contextualize the clinical observations presented in this mechanistic case series rather than serving as a systematic review of DTI.

DTI is classically defined as pressure-related tissue damage involving underlying soft tissues beneath intact or discolored skin, typically occurring over bony prominences [9]. However, not all clinically observed lesions conform fully to a purely pressure-based model. In perioperative settings, particularly in the gluteal region, lesions may develop with delayed onset, absence of overt cutaneous ulceration, and biochemical or radiological evidence of deep muscle injury. These observations suggest that mechanical pressure alone may not fully explain DTI pathophysiology in some clinical contexts.

S2, 2. Ischemia–Reperfusion Injury: Core Mechanisms

Ischemia–reperfusion injury refers to tissue damage occurring following blood flow restoration after ischemia. This process involves several interrelated mechanisms:

- Accumulation of metabolic byproducts and tissue acidosis during ischemia
- Sudden oxygen influx upon reperfusion leading to increased reactive oxygen species (ROS) generation
- Disruption of mitochondrial function and intracellular calcium homeostasis
- Activation of inflammatory pathways causing endothelial and membrane injury

These mechanisms have been extensively described in diverse clinical settings, including myocardial infarction,

organ transplantation, and skeletal muscle compression injury [11] [12] [18].

S2, 3. Potential Links Between DTI and Ischemia–Reperfusion Processes

S2, 3.1. Intraoperative Immobility and Gluteal Muscle Ischemia

During prolonged surgical procedures under general or neuraxial anesthesia, patients experience motor and sensory blockade that limits spontaneous repositioning. Sustained gluteal pressure may compromise capillary perfusion [11] [25], potentially causing localized hypoxia and metabolic disturbance within deep muscle tissues.

S2, 3.2. Delayed Erythema as a Possible Manifestation of Reperfusion

In some cases, visible skin changes, such as deep non-blanchable erythema, appear 12–24 h postoperatively. This temporal pattern is consistent with reperfusion-phase processes [12] [18], during which ROS generation, endothelial dysfunction, and microvascular leakage may lead to edema and discoloration.

S2, 3.3. Muscle Enzyme Elevation and Imaging Correlates

Elevation of muscle-associated enzymes, including creatine kinase (CK), aspartate aminotransferase (AST), and lactate dehydrogenase (LDH), within 24–48 h post-surgery suggests underlying muscle injury. Imaging modalities such as CT or MRI frequently demonstrate gluteal muscle edema without epidermal disruption, findings compatible with subdermal tissue injury [10] [11].

S2, 4. Supporting Literature and Experimental Observations

A growing body of literature supports the possibility that ischemia–reperfusion processes contribute to DTI development. Wynn et al. (2022) described DTI as potentially involving hypoxic reperfusion–related mechanisms [10], emphasizing oxidative and microvascular injury beyond mechanical loading. Experimental animal studies have demonstrated irreversible muscle damage following release of sustained pressure [11] [12] [18], characterized by ROS accumulation, endothelial injury, and inflammatory cell infiltration. Similar pathophysiological patterns have been observed in clinical perioperative DTI reports [9] [10].

S2, 5. Conclusion

DTI in perioperative settings may, in some clinical contexts, involve ischemia–reperfusion–related mechanisms in addition to sustained mechanical loading. Recognition of this broader pathophysiological framework may assist in

refining diagnostic interpretation, informing preventive strategies aimed at minimizing prolonged ischemia and reperfusion stress, and improving clinical understanding of postoperative soft tissue injuries.

References (Supplementary Materials)

4. Ono, T. Conditions under which electrosurgical burns occur. *Clin. Eng.* **2008**, *19*, 959–965. (In Japanese).

9. National Pressure Injury Advisory Panel (NPIAP); European Pressure Ulcer Advisory Panel (EPUAP); Pan Pacific Pressure Injury Alliance (PPPIA). Prevention and Treatment of Pressure Ulcers/Injuries: Clinical Practice Guideline—The International Guideline; EPUAP/NPIAP/PPPIA: 2019. Available online:https://static1.squarespace.com/static/6479484083027f25a6246fcb/t/6553d3440e18d57a550c4e7e/1699992399539/CPG2019edition-digital-Nov2023version.pdf (accessed on 11 February 2026).

10. Wynn, M.; Stephens, M.; Pradeep, S.; Kennedy, R. Risk factors for the development and evolution of deep tissue injuries: A systematic review. *J. Tissue Viability* **2022**, *31*, 416–423. https://doi.org/10.1016/j.jtv.2022.03.002.

11. Loerakker, S.; Manders, E.; Strijkers, G.J.; Nicolay, K.; Baaijens, F.P.T.; Bader, D.L.; Oomens, C.W.J. The effects of deformation, ischemia, and reperfusion on the development of muscle damage during prolonged loading. *J. Appl. Physiol.* **2011**, *111*, 1168–1177. https://doi.org/10.1152/japplphysiol.00389.2011.

12. Peirce, S.M.; Skalak, T.C.; Rodeheaver, G.T. Ischemia–reperfusion injury in chronic pressure ulcer formation: A skin model in the rat. *Wound Repair Regen.* **2000**, *8*, 68–76. https://doi.org/10.1046/j.1524-475x.2000.00068.x

18. Tsuji, S.; Ichioka, S.; Sekiya, N.; Nakatsuka, T. Analysis of ischemia–reperfusion injury in a microcirculatory model of pressure ulcers. *Wound Repair Regen.* **2005**, *13*, 209–215. https://doi.org/10.1111/j.1067-1927.2005.130213.x.

23. Koyama, Y.; Hayashi, N. Electrical safety management in operating rooms. *Clin. Eng. (JP.).* **2008**, *19*, 953–958.

24. *IEC 60601-2-2*; Medical Electrical Equipment—Particular Requirements for the Basic Safety and Essential Performance of High-Frequency Surgical Equipment. International Electrotechnical Commission: Geneva, Switzerland, 2017.

25. Gefen, A. Bioengineering models of deep tissue injury. *Adv. Skin Wound Care* **2008**, *21*, 30–36. https://doi.org/10.1097/01.ASW.0000305403.89737.6c
